# Placental erythropoietin expression is upregulated in growth-restricted fetuses with abnormal umbilical artery Doppler findings: a case–control study of monochorionic twins

**DOI:** 10.1186/s12884-018-1963-2

**Published:** 2018-08-08

**Authors:** Yao-Lung Chang, An-Shine Chao, Hsiu-Huei Peng, Shuenn-Dyh Chang, Kuan-Ju Chen, Po-Jen Cheng, Tzu-Hao Wang

**Affiliations:** 1 Department of Obstetrics and Gynecology, Chang Gung Memorial Hospital, Linkou, College of Medicine, Chang Gung University, No.5, Fu-Shin Road, Gwei-Shan District Taoyuan City, Taiwan; 2 School of Traditional Chinese Medicine, College of Medicine, Chang Gung University, No.5, Fu-Shin Road, Gwei-Shan District Taoyuan City, Taiwan; 3 Genomic Medicine Research Core Laboratory (GMRCL), Chang Gung Memorial Hospital, No.5, Fu-Shin Road, Gwei-Shan District Taoyuan City, Taiwan

**Keywords:** Erythropoietin, Monochorionic twin, Intrauterine growth restriction, Placenta

## Abstract

**Background:**

We previously reported that fetal plasma erythropoietin (EPO) concentrations are significantly increased in growth-restricted fetuses with abnormal umbilical artery (UA) Doppler. During hypoxia in an ovine model, the primary site of fetal EPO synthesis was switched from the kidneys to the placenta. Therefore, we designed this study to evaluate human placental EPO gene expression and the correlation to fetal serum EPO concentration in growth-restricted fetuses in a monochorionic (MC) twin model.

**Methods:**

In MC twin pairs, selective intrauterine growth restriction (sIUGR) was defined as the presence of (i) birth weight discordance of > 20% and (ii) a smaller twin with a birth weight less than the 10th percentile. Fetal UA and middle cerebral artery (MCA) Doppler were checked within 1 week before delivery. An abnormal UA Doppler was defined as persistently absent or reverse end-diastolic flow. Cerebroplacental ratio (CPR) was defined as MCA-pulsatility index (PI)/UA-PI. Fetal plasma EPO concentrations were measured in cord blood, and EPO gene expression was assayed in each twin’s placental territory. The intertwin plasma EPO ratio was calculated as the cord plasma EPO level of the smaller (or sIUGR) twin divided by the EPO concentration of the larger (or appropriate-for-gestational-age (AGA)) twin, and the intertwin placental EPO gene expression ratio was calculated similarly.

**Results:**

Twenty-six MC twins were analyzed, including normal twins (Group 1, *n* = 9), twins with sIUGR without UA Doppler abnormalities (Group 2, *n* = 9), and twins with sIUGR and UA Doppler abnormalities (Group 3, *n* = 8). The CPRs of smaller (sIUGR) fetuses were significantly decreased in Group 3 MC twins (*p* < 0.001), but not significantly different between Group 1 and Group 2. The highest fetal plasma EPO ratio and placental EPO gene expression ratio were identified in Group 3 MC twins (*p* < 0.001). The placental EPO gene expression ratios were significantly correlated with the fetal plasma EPO ratios (Pearson’s correlation test, *p* = 0.004).

**Conclusion:**

This study provides evidence of increased placental EPO expression in MC twin fetuses with sIUGR and abnormal UA Doppler. Future studies are needed to confirm the similar role of placental EPO in severe IUGR singletons.

## Background

In both fetuses and adults, erythropoietin (EPO) secretion is stimulated by low oxygen level [1]. In adults, EPO is produced predominantly by the kidneys. In human fetuses with congenital absence of bilateral kidneys [2] and animal fetuses with surgical nephrectomy [3, 4], plasma EPO and hematocrit concentrations remain normal, indicating that the fetal kidneys are not the only site for EPO production. In an ovine study of artificially induced maternal hypoxia, the primary site of fetal EPO synthesis was switched from the kidneys to the placenta [5]. Because EPO does not cross the placenta in humans [6], maternal EPO is not a source of EPO for the fetus.

MC twins share an identical genetic makeup and maternal physiology, but unequal placental sharing has been shown to result in the development of sIUGR [7, 8] . SIUGR, defined as the presence of IUGR in one twin while the other is appropriate for gestational age (AGA), occurs in 12%–15% of all MC twin pregnancies [7]. According to the UA Doppler findings of the IUGR twin [9], MC twins with selective IUGR were classified as three types: (1) normal with positive diastolic velocity, (2) persistent absent or reverse of end-diastolic velocity (AREDV) and (3) intermittent AREDV. When umbilical artery (UA) Doppler findings for sIUGR twins revealed persistent or intermittent UA-AREDF, the risk of intrauterine fetal demise was reported to increase [7, 9].

We have previously reported that fetal plasma EPO concentrations were significantly increased in monochorionic (MC) twins with selective intrauterine growth restriction (sIUGR) and umbilical artery Doppler abnormality [10]. Because the two fetuses in an MC twin pregnancy share an identical genetic makeup, the fetal plasma EPO concentrations of the AGA fetus can serve as an internal control for the corresponding levels detected in the sIUGR twin. In this setting, the detection of significant differences in fetal EPO concentrations in MC twin pregnancies with sIUGR and abnormal UA Doppler (accompanied by the lack of such differences in MC twin pregnancies without sIUGR) suggests changes in fetal plasma EPO might be associated with growth restriction and abnormal UA Doppler.

Since the contribution of the human placenta to fetal plasma EPO concentrations during hypoxia remains unclear, to fill this knowledge gap, we conducted this study to analyze the human placental EPO gene expression and correlate placental EPO expression ratios to fetal plasma EPO ratios in growth-restricted fetuses in a MC twin model.

## Methods

This study was conducted from October 2014 to May 2016 at Chang Gung Memorial Hospital, Linkou Medical Center, Taiwan. The study protocol was approved by the Institutional Review Board of the Chang Gung Medical Foundation (IRB #101-4803A3). The inclusion criteria required that MC twins were delivered in our hospital, mothers with MC twins provided informed consent, and cord blood and placental tissues were successfully collected. The exclusion criteria were the presence of twin-to-twin transfusion syndrome (TTTS), monoamniotic twins, twin anemia–polycythemia sequence (TAPS), and congenital, structural or genetic malformations in the fetus. Because uterine contractions may modify the fetal plasma EPO levels [1], only MC twins delivered through cesarean section before the beginning of labor were included.

The diagnosis of TTTS was based on the Quintero criteria [11]. TAPS was diagnosed in the presence of a postnatal intertwin hemoglobin difference of > 8 g/dL [12]. During the first trimester or early second trimester, MC twin pregnancies were identified using the following ultrasonographic criteria: (i) the presence of a single placenta, (ii) the presence of a thin dividing membrane, and (iii) the absence of a twin peak (lambda) sign. Monochorionicity was confirmed by obstetricians through postpartum examination of the placenta (presence of a single placenta with intertwin anastomoses).

### Measurements of pulsed-wave Doppler in umbilical artery (UA) and middle cerebral artery (MCA), and calculation of cerebroplacental ratio (CPR)

Pulsed-wave Doppler UA and MCA studies were performed using GE Voluson 730 Expert ultrasound machines within seven days before delivery; if more than one examination data are available then the last examination data were collected. Measurements were obtained during fetal quiescence. Means of three consecutive automated measurements were recorded for analysis for each fetus at each examination. A mid-segment of a free loop of umbilical cord was chosen as the site of measurement. Persistent UA-AREDF was recognized as abnormal UA Doppler, whereas intermittent UA-AREDF was recognized as normal UA Doppler. Measurements of the MCA indices were performed in the proximal one third of the MCA at the vessel nearest the transducer with the angle of insonation close to 0°. CPR is defined as: MCA-pulsatility index (PI)/UA-PI.

In this study, sIUGR was also defined as the presence of (i) a birth weight discordance of > 20% and (ii) a smaller twin with a birth weight less than the 10th percentile [13, 14]. Birth weight discordance was calculated as the difference between the fetal weights of the larger and smaller twins divided by the fetal weight of the larger twin, as follows: [(body weight of the AGA or larger twin − body weight of the sIUGR or smaller twin)/(body weight of the AGA or larger twin)] × 100%. An abnormal UA Doppler was defined as persistent UA-AREDF. MC twins without sIUGR were defined as Group 1, those with sIUGR but without UA Doppler abnormalities as Group 2, and those with both sIUGR and UA Doppler abnormalities as Group 3.

### Collection of fetal blood

After delivery of the fetus during cesarean section, the umbilical cord of each baby was doubly clamped. Before removing the placenta, surgeons on the operating table collected cord blood from the placenta by puncturing the umbilical vein. Cord blood was sent to the Department of Clinical Pathology for EPO measurements using a commercially available chemiluminescent immunometric assay (IMMULITE® 2000 systems, Siemens, Los Angeles, USA). The intertwin EPO ratio was defined as the plasma EPO concentration of the sIUGR twin divided by the EPO concentration of the AGA twin.

### Inspection and collection of placental tissues

Placentas were collected immediately after delivery. After the blood was drained from the umbilical vessels, the placenta was washed with ice-cold phosphate-buffered saline to remove all blood clots and was examined for the integrity of the cotyledons and membranes. The vascular equator was defined as a border drawn in the middle of the vascular zone on the chorionic fetal surface where the communicating vessels met. The placentas were cut along the vascular equator. We collected two to three pieces of fresh placental tissue (0.5 × 0.5 × 0.5 cm) from the placental territory of each MC twin. The placental biopsies were taken from the middle layer of the placenta between the maternal and fetal surfaces. Regions with obvious calcification or infarction were avoided. The placental specimens were briefly rinsed with normal saline to remove the blood. The placental tissues were kept in RNALater reagent (Thermo Fisher Scientific, Waltham, MA, USA) at 4 °C for 24–48 h. Subsequently, the tissues were transferred to new tubes and kept in a freezer at − 70 °C for long-term storage [15]. EPO gene expression was measured in each twin’s placental territory.

### Placental EPO gene expression analyses

Total RNA was isolated from placental tissues by using TRIzol reagent (Invitrogen), according to the manufacturer’s instructions. Complementary DNA (cDNA) synthesis was performed as per the manufacturer’s instructions by using the SuperScript III first-strand cDNA synthesis system (Invitrogen). Quantitative real-time (RT)-PCR was performed using the SYBR Green method (Invitrogen). Placental EPO expression was analyzed in duplicate and normalized to the GAPDH values by using the following primer sequences: EPO sense: 5′-caaggaggccgagaatatca-3, EPO antisense: 5′-tccatcctcttccaggcata-3′, GAPDH sense: 5′-GGTATCGTGGAAGGACTCATGAC-3′, GAPDH antisense: 5′-ATGCCAGTGAGCTTCCCGT-3′. RT-PCR data are presented as △Ct (target gene expression normalized to the housekeeping gene) and the fold changes in expression [16]. The placental EPO gene expression ratio for the two placental territories in a twin pair is presented as the fold changes between the two fetuses in MC twins: (relative EPO expression levels of the smaller (sIUGR) twin)/ (relative EPO expression levels of the larger (AGA) twin).

### Data analysis and statistics

Data were analyzed using SPSS 11.0 statistical software (SPSS Inc., Chicago, IL, USA). Intergroup comparisons of continuous variables among the three study groups were performed using one-way analysis of variance (ANOVA), followed by post hoc at least significance difference (LSD) tests for multiple comparisons. Paired Student’s t tests were used to compare continuous data within each pair. The correlations between the plasma EPO ratio and placental EPO gene expression ratio were analyzed using Pearson’s correlation coefficients. Two-tailed *p* values of < 0.05 were considered statistically significant.

## Results

Twenty-six MC twin pairs met the inclusion criteria and were selected for this study. They were classified as Group 1 (*n* = 9), normal MC twins; Group 2 (*n* = 9), MC twins with sIUGR but without UA Doppler abnormalities; and Group 3 (*n* = 8), MC twins with sIUGR and UA Doppler abnormalities.

There was no significant difference in maternal age at delivery among the three groups of MC twins. MC twins in Group 3 were delivered significantly earlier (mean: 30.7 weeks of gestation) than those in Groups 1 and 2. The birth weights of both AGA and sIUGR twins were lowest in Group 3 and highest in Group 1; likely reflecting the shortest gestational age of delivery in Group 3 and longest in Group 1. The observed birth weight discordance was most severe in Group 3, mainly due to the lowest birth weights being observed in the sIUGR fetuses of this group. The maternal body mass indices (BMIs) were not significantly different among the three groups of MC twins. No significant difference was observed in the hemoglobin levels between the two fetuses in any of the groups of MC twins (Table 1).

**Table 1:** Characteristics of three groups of monochorionic twin pregnancies

	Group 1 (*n* = 9)	Group 2 (*n* = 9)	Group 3 (*n* = 8)	*p* value
Maternal age at delivery	31.4 ± 6.2	31.1 ± 3.2	32.4 ± 2.2	0.80^#^
Gestational age of delivery (weeks)	36.7 ± 0.97	34.8 ± 1.7	30.7 ± 2.7	< 0.001^#^(Group1 > Group2 > Group 3)
Birth weight of the AGA (larger) twin (g)	2597 ± 271	2237 ± 395	1398 ± 403	< 0.001^#^(Groups 1 > Group 2 > Group 3)
Birth weight of the sIUGR (smaller) twin (g)	2333 ± 276	1573 ± 300	717 ± 232	< 0.001^#^(Groups 1 > Group 2 > Group 3)
Birth weight discordance (%)	10.0 ± 6.6	29.5 ± 7.4	48.9 ± 7.4	< 0.001^#^(Groups 3 > Group 2 > Group 1)
Cord plasma EPO-ratio	1.03 ± 0.20	1.36 ± 0.76	1.95 ± 0.59	0.01^#^(Group3 > Groups 1 and 2)
Cord plasma EPO of larger (AGA) fetus (mIU/ mL)	14.2 ± 11.4	10.5 ± 9.2	24.7 ± 36.8	0.410^#^
Cord plasma EPO of smaller (sIUGR) fetus (mIU/ mL)	16.0 ± 14.6	12.7 ± 9.3	49.4 ± 71.8	0.153^#^
Maternal BMI at delivery	26.6 ± 1.9	27.4 ± 3.4	27.1 ± 3.2	0.880^#^
CPR of larger (AGA) fetus	1.39 ± 0.21	1.47 ± 0.29	1.74 ± 0.29	0.064^#^
CPR of smaller (sIUGR) fetus	1.41 ± 0.20	1.33 ± 0.13	0.45 ± 0.03	< 0.001^#^(Group3 < Groups 1 and 2)
Neonatal hemoglobin levels (larger/AGA) (g/dL)	14.8 ± 1.2	16.4 ± 1.8	16.8 ± 1.1	※
Neonatal hemoglobin levels (smaller/sIUGR) (g/dL)	14.4 ± 1.2	16.5 ± 2.3	15.6 ± 1.7	※

Group 1: normal MC twins; group 2: twins with sIUGR without UA Doppler abnormalities; group 3: twins with sIUGR and UA Doppler abnormalities

Birth-weight discordance: [(body weight of the larger or AGA twin - body weight of the smaller or sIUGR twin) / body weight of the larger or AGA twin] × 100%

Cord plasma EPO-ratio: [plasma EPO level of the smaller (or the small-for-gestational-age) twin] / [plasma EPO concentration of the larger (or the appropriate-for-gestational-age) twin]

CPR (cerebroplacental ratio) = (middle cerebral artery-pulsatility index) / (umbilical artery- pulsatility index)

Abbreviations: *MC* monochorionic, *sIUGR* selective intrauterine growth restriction, *AGA* appropriate-for-gestational-age, *EPO* erythropoietin, *CPR* cerebroplacental ratio

^#^One-way analysis of variance

※No significant difference was observed in the hemoglobin levels between the two fetuses in any of the groups of MC twins

**Fig. 1:**
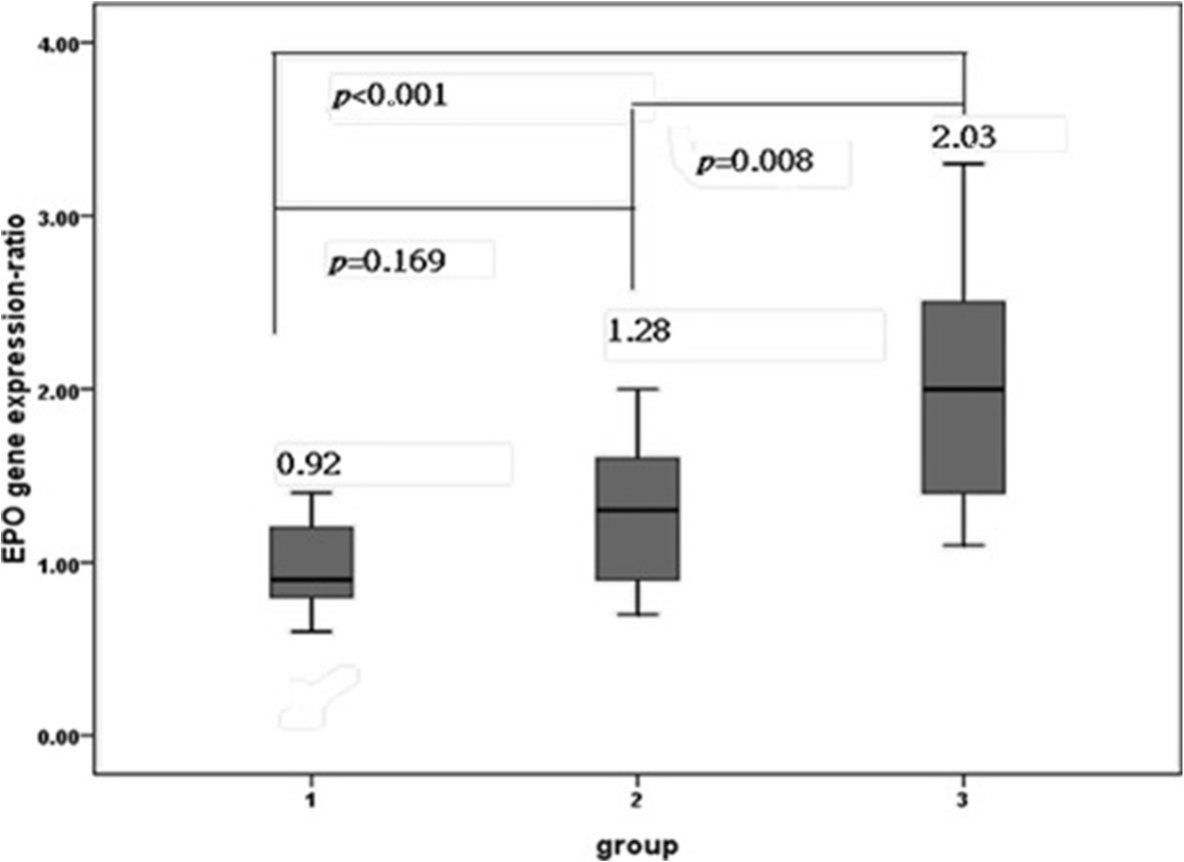
Placental EPO gene expression ratios in three groups of MC twins. The placental EPO gene expression ratio was significantly higher in Group 3 (one-way ANOVA, *p* < 0.001), but no significant differences were observed between Groups 1 and 2 (post hoc comparison using the LSD test)**.** The placental EPO gene expression ratios for the two placental territories in a twin pair are presented as fold changes between the two fetuses in MC twins: (the relative EPO expression concentrations of the sIUGR twin)/ (relative EPO expression concentrations of the larger twin). A higher EPO gene expression ratio (as shown in group 3) indicated more EPO expression by the placental territory of the sIUGR twin. Data are expressed as means and ranges of values. Group 1: normal MC twins; Group 2: twins with sIUGR but without UA Doppler abnormalities; and Group 3: twins with sIUGR and UA Doppler abnormalities. Abbreviations: EPO: erythropoietin; MC: monochorionic; sIUGR: selective intrauterine growth restriction; AGA: appropriate for gestational age; UA: umbilical artery; LSD: least significant difference

**Fig. 2:**
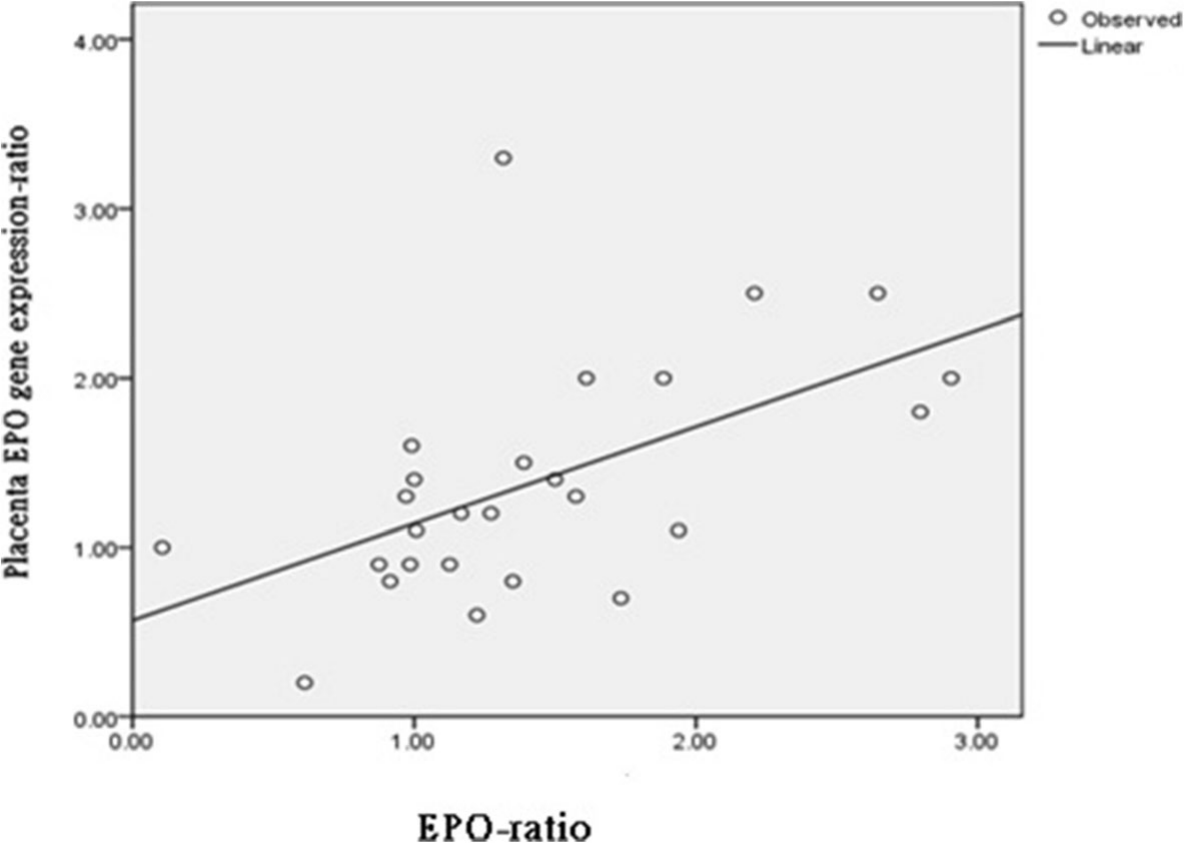
Positive correlations between the cord plasma EPO ratios and placental EPO gene expression ratios. The cord plasma EPO ratio is plasma EPO concentration in the smaller (or the small-for-gestational-age) twin divided by the EPO concentration of the larger (or the appropriate-for-gestational-age) twin. The placental EPO gene expression ratios for the two placental territories in a twin pair are presented as fold changes between the two fetuses in MC twins: (relative EPO expression concentrations of the sIUGR twin) / (relative EPO expression concentrations of the AGA twin). Abbreviations: EPO: erythropoietin; MC: monochorionic; sIUGR: selective intrauterine growth restriction; AGA: appropriate for gestational age

The placental EPO gene expression ratios in Group 3 were significantly higher than those in Groups 1 and 2 MC twins, but no significant differences were noted between the twin pregnancies in Groups 1 and 2 (Fig. 1). The fetal plasma EPO ratios in Group 3 were significantly higher than those in Groups 1 and 2 (Table 1). Cerebroplacental ratios (CPRs) derived from MCA and UA pulsatility index (PI) were not significantly different among larger (AGA) fetuses in three groups of MC twins. However, CPRs of the smaller (sIUGR) fetuses were significantly decreased in groups 3 MC twins, but not significantly different between group 1 and 2 MC twins (Table 1). The placental EPO gene expression ratios were significantly correlated with the fetal plasma EPO ratios (Pearson’s correlation test, *p* = 0.004**;** Fig. 2**).**

## Discussion

This study reports that the placental expression of EPO in human twin pairs is associated with fetal growth restriction and abnormal UA Doppler. We did not include TTTS and TAPS in this study, so we could examine individual placental gene expression without interference by communicating blood vessels between twins. Both placental territories of MC twins share an identical genetic makeup, but the sIUGR fetus with abnormal UA Doppler findings has a smaller placental territory and receives less placental perfusion [7, 8]. Therefore, we hypothesized that hypoperfusion to the severe sIUGR fetus may stimulate its placental EPO expression. Our current results (Table 1**,** Figs. 1 and 2) support this hypothesis.

Group 3 MC twins were delivered earliest. Given the lack of significant correlations between the gestational age at delivery and plasma EPO concentrations in the sIUGR or AGA fetuses [10], we could hypothesize that the increased cord plasma EPO ratios and placental EPO gene expression ratios in Group 3 MC twins may not be caused by early delivery. Notably, good correlations were observed between the cord plasma EPO ratios and placental EPO expression ratios (Fig. 2), strongly supporting our hypothesis that the upregulated placental EPO gene expression in Group 3 MC twins accounts for the increased fetal plasma EPO concentrations in fetuses with hypoperfusion. The placenta has been long considered as a neuroendocrine organ [17, 18], and neuroepithelial cells have been shown to produce EPO in the earliest stage of life in mouse embryos [19]. These reports collectively support the notion that the placenta can be the site of EPO generation.

In addition to stimulating the production of red blood cells, EPO was recently shown to have functions such as inhibition of apoptosis and stimulation of inflammatory and proangiogenic mechanisms in multiple organ systems [20]. These nonerythroid functions of EPO have been considered to protect tissues from hypoxia and ischemia/reperfusion injury [21, 22]. The pleiotropic cytokine EPO was reported to significantly attenuate brain injury induced by repeated lipopolysaccharide (LPS) exposure [23]. Treatment with recombinant human EPO can also ameliorate LPS-induced damage to the placenta and fetal liver [24]. In this study, no significant differences were observed in the hemoglobin levels between the AGA and sIUGR fetuses in Group 3. Therefore, the increase in fetal plasma EPO concentrations is unlikely to only promote erythropoiesis; instead, it may have a role in tissue protection from decompensated hypoxia in sIUGR fetuses.

In fetuses with chronic hypoxia, the UA-PI increase but MCA-PI decrease, reflecting a brain sparing process [25]. The ratio of MCA-PI to UA-PI, named as the cerebroplacental ratio (CPR), has been shown to be a better proxy marker for fetal compromise during placental insufficiency and hypoxemia. Discordant CPR is an independent predictor for the risk of perinatal loss in twin pregnancies [26]. In group 3 MC twins, the sIUGR fetuses had the lowest CPRs, indicating that group 3 sIUGR fetuses had the severest insufficiency of placental perfusion. These results support that the higher placental EPO expression ratio and the higher fetal plasma EPO concentration ratio may be caused by malperfusion to the placental territory of the sIUGR fetus.

The use of MC twin models is instrumental in clearly identifying the increased EPO expression levels in the less perfused placenta, because an MC twin model provides identical genetic and environmental conditions, minimizing individual, basal noises in gene expression. Thus, analysis of MC twins can reveal subtle changes that are impossible to observe in other pregnant human case–control studies.

The limitations of this study are as follows. First, the histological site of EPO production in the human placenta could not be identified, although EPO mRNA has been detected at the apical tips of the fetal villi in the inner margin of the caruncle [27]. Second, we could not quantify the proportion, if not all, of the fetal plasma EPO amount derived from the placenta in the setting of uncompensated hypoxia. However, the placental EPO gene expression ratios were well correlated with the fetal plasma EPO ratios. Third, individual disease severity forced us to deliver twin babies at different ages of gestation, resulting in a significant difference in the gestational ages at delivery and birth weight of the AGA twins among the three groups. Despite our endeavors to minimize such confounding age factors by only comparing inter-twin differences in each pair of twins (Fig. 1), we still cannot completely rule out that differences in gestational age do not affect cord plasma EPO ratios at all. Finally, because many women in this study were referred by other hospitals, we lacked information regarding the pregestational BMI and maternal body weight gain during pregnancy. Therefore, we could not evaluate the potential involvement of placental lipotoxic environments induced by maternal obesity [28].

## Conclusion

Results of this MC twin study provide human evidence of increased placentalEPO expression in sIUGR fetuses with abnormal UA Doppler. The observed increased EPO expression in this group is correlated with increased plasma EPO concentrations.

## Abbreviations

AGA: Appropriate for gestational age
ANOVA: Analysis of variance
AREDF: Absent or reverse end-diastolic flow
CPR: Cerebroplacental ratio
EPO: Erythropoietin
LPS: Lipopolysaccharide
LSD: Least significance difference
MC: Monochorionic
MCA: Middle cerebral artery
PI: Pulsatility index
rhEPO: recombinant human erythropoietin
sIUGR: selective intrauterine growth restriction
TAPS: Twin anemia–polycythemia sequence
TTTS: Twin–twin transfusion syndrome
UA: Umbilical artery
